# Mixed Capillary Venous Retroperitoneal Hemangioma

**DOI:** 10.1155/2013/258352

**Published:** 2013-02-28

**Authors:** Mohit Godar, Qinghai Yuan, Rukeshman Shakya, Yang Xia, Pengguo Zhang

**Affiliations:** ^1^Department of Radiology, The Second Hospital of Jilin University, Norman Bethune College of Medicine, 218 Ziqiang Street, Nanguan District, Changchun, Jilin 130041, China; ^2^Department of Pathology, The Second Hospital of Jilin University, Norman Bethune College of Medicine, 218 Ziqiang Street, Nanguan District, Changchun, Jilin 130041, China

## Abstract

We report a case of mixed capillary venous hemangioma of the retroperitoneum in a 61-year-old man. Abdominal ultrasonography showed a mass to be hypoechoic with increased flow in color Doppler imaging. Dynamic contrast-enhanced computed tomography revealed a centripetal filling-in of the mass, located anterior to the left psoas muscle at the level of sacroiliac joint. On the basis of imaging features, preoperative diagnosis of hemangioma was considered and the mass was excised by laparoscopic method. Immunohistochemical studies were strongly positive for CD31 and CD34, and negative for calretinin, EMA, WT1, HMB45, Ki67, synaptophysin, and lymphatic endothelial cell marker D2–40. Histologically, the neoplasm was diagnosed as mixed capillary venous hemangioma.

## 1. Introduction

Primary retroperitoneal hemangioma is an extremely rare entity, of which, the most common is cavernous hemangioma. We report a case of retroperitoneal hemangioma which was diagnosed preoperatively by contrast-enhanced scan performed on a 256-slice multidetector computed tomography (CT). This is the first case report to our knowledge that describes a mixed capillary venous hemangioma in the retroperitoneal space.

## 2. Case Presentation

**Figure 1:**
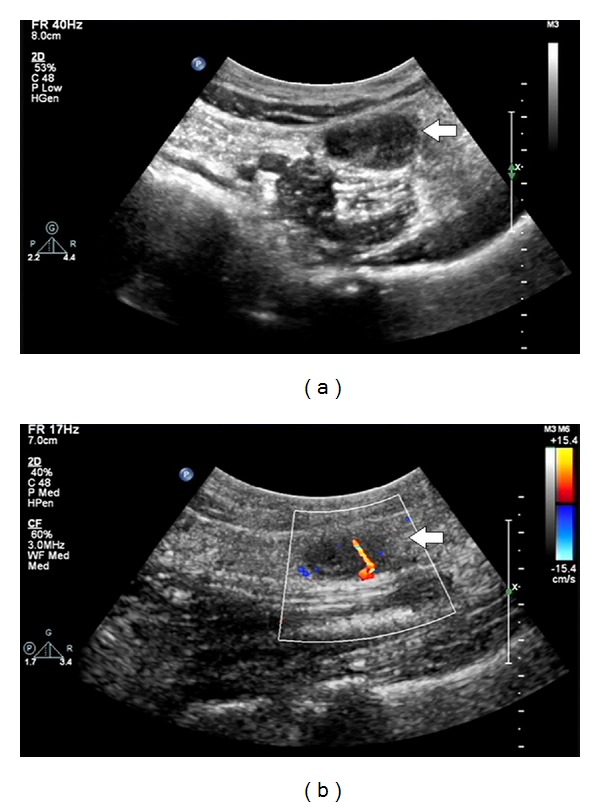
Grey-scale ultrasonography shows (a) a mild heterogenous hypoechoic mass with posterior acoustic enhancement anterior to the left psoas muscle with increased flow within the mass on color Doppler imaging (b) (arrow).

**Figure 2:**
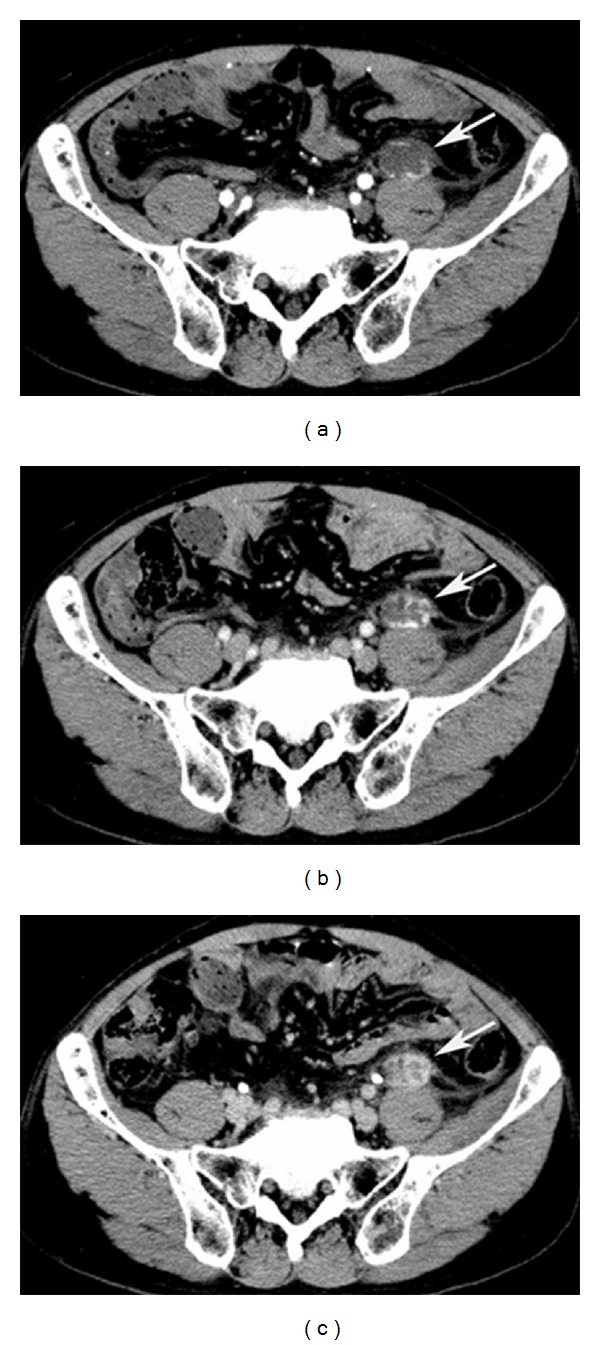
Dynamic contrast enhanced computed tomography. During the arterial phase (a), lesion demonstrates an initial peripheral nodular, with subsequent centripetal progressive filling-in pattern during venous (b) and equilibrium phase (c) (arrow).

**Figure 3:**
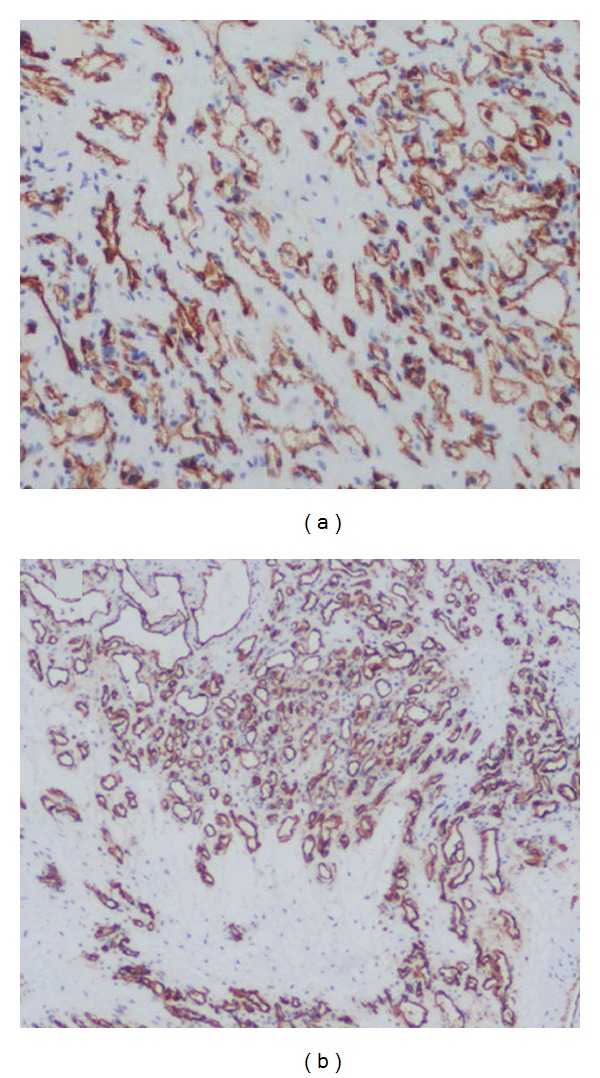
Diffuse immunoreactivity of tumor cells to CD31 (a) and CD34 (b) confirms an endothelial origin.

**Figure 4:**
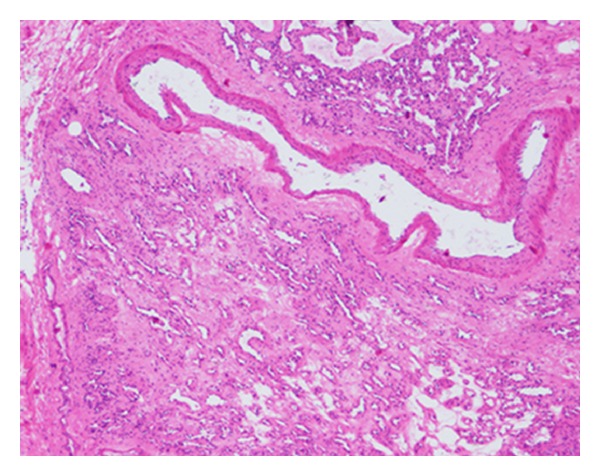
Histopathologic examination of the resected specimen. The neoplasm is composed of vein with thick muscular wall and proliferation of small capillary-sized vessels (hematoxylin and eosin, ×40).

A 61 year-old otherwise healthy male presented with complains of intermittent, dull lower left abdominal pain, and back discomfort for 2 months. The patient has no other associated symptoms. A physical examination revealed a firm, mobile palpable mass 2 cm × 2 cm in size in the left lower quadrant of abdomen, associated with mild tenderness. Laboratory and biochemical examinations were reported normal. Abdominal ultrasonography (US) (performed on iE-33 system, Philips, Bothell, WA) showed a round, well-defined, mild heterogenous hypoechoic mass with posterior acoustic enhancement, anterior to the left psoas muscle with increased flow on color Doppler imaging (Figure 1). Dynamic contrast-enhanced scan performed on a 256-slice multidetector CT scanner (Brilliance iCT 256; Philips Healthcare) showed a round, well-defined, low density 3 cm × 1.8 cm soft-tissue mass, anterior to the left psoas muscle at the level of left sacroiliac joint with peripheral nodular enhancement and progressive filling of the mass (Figure 2). Based on the CT findings, preoperative diagnosis of hemangioma was considered and the patient underwent surgery. A soft, ill-defined yellowish tumor, measuring 3 cm × 2 cm with focal dilated spaces, was excised by laparoscopic method. Immunohistochemically, the tumor cells were diffusely positive for vascular endothelial markers CD31 and CD34 and negative for calretinin, EMA, WT1, HMB45, Ki67, synaptophysin, and lymphatic endothelial cell marker D2–40 (Figure 3). Histopathologically, the diagnosis was confirmed as mixed venous capillary retroperitoneal hemangioma (Figure 4). Postoperative period was uneventful and the patient was discharged 9 days after the surgery.

## 3. Discussion

Primary retroperitoneal tumors are relatively rare entity and constitute 0.07% to 0.6% of all tumors and around 62–82% are malignant, with liposarcoma being most common [1]. Benign retroperitoneal neoplasms are uncommon, and vascular tumor such as hemangioma is rather exceptional, with only a few cases being reported [1–5]. Among the reported cases of retroperitoneal hemangiomas, cavernous is the most common, whereas venous hemangioma is unusual [1–3]. In the present case, hemangioma was admixture of capillary and venous, which has never been reported previously in the retroperitoneum. Most hemangiomas are present at birth which rapidly proliferate and thereafter slowly involute and regress completely, while others grow more rapidly during puberty, pregnancy, or following trauma. The growth in size of the hemangioma is the result of thrombosis or obstruction of the blood flow [1, 2, 5]. Symptoms secondary to retroperitoneal hemangioma depend upon the location and size of the tumor. Since the retroperitoneal space is vast and away from critical organs, symptoms are usually vague and nonspecific until tumor attains large size compressing or invading adjacent structure [6]. Typically, retroperitoneal hemangiomas have been reported to be firmly attached to spleen [2], peritoneum, kidney, nearby vessels [3], and in our case, to the left psoas muscle [3]. Therefore, surgeons should pay attention to intraoperative bleeding and bleeding associated with removal of the lesion [2]. 

Although US is helpful in differentiating cystic from solid tumors, US appearance and echogenicity of hemangioma can be variable, from hyperechoic to anechoic, typically with posterior acoustic enhancement. CT images of previously reported cases showed cystic mass with minor or poorly enhanced lesion [3] or calcified ring [6]. In the present case dynamic contrast CT was typical for previously reported hemangioma [7], peripheral nodular enhancement in arterial phase and with the centripetal enhancement of the mass in venous and equilibrium phase, which prompt us to make the diagnosis preoperatively. Nonetheless, characteristic imaging findings of hemangiomas are not always possible and atypical CT findings can be due to neovascularity, arteriovenous shunting, thrombosis, and hemorrhage that decrease blood flow with delay in filling up of contrast [5]. CT enhancement can be seen in lymphangioma, hemangiopericytoma, and malignant fibrous histiocytoma and should be considered for differential diagnosis [6]. Preoperative diagnosis of retroperitoneal is unusual; only one reported case based on characteristic MRI finding with low signal intensity on the T1-weighted image and high signal intensity on the T2-weighted image with partial interruption of the muscle layer surrounding the tumor [2]. However, even if a hemangioma was suspected by MRI, the differential diagnosis between venous hemangiomas and cavernous or capillary hemangiomas appeared to be difficult [2].

Virtually there is no risk of malignant transformation of hemangioma. Surgical treatment is usually indicated for large size tumor that has risk of rupture or bleeding [1, 2]. While size of the hemangioma in our case was relatively small, the main indication of surgery was recurrent pain, and also the majority of retroperitoneal tumors are malignant requiring histopathological diagnosis. Immunohistochemically, hemangiomas are positive for vascular endothelial markers CD31, CD34 and factor VIII, whereas lymphatic endothelial cell marker D2–40 is negative, which essentially excludes lymphangioma. Recurrence after complete resection is rare with one previously reported case of recurrence probably due to incomplete resection [3]. 

In conclusion, we highlight a case of a benign vascular neoplasm in the retroperitoneal space. Although retroperitoneal hemangioma is rare entity, it should be considered in the differential diagnosis of retroperitoneal mass. While imaging feature of hemangioma can be typical occasionally, it should be correlated with histology to make a correct diagnosis.
